# Opening the ‘black box’ of collaborative improvement: a qualitative evaluation of a pilot intervention to improve quality of malaria surveillance data in public health centres in Uganda

**DOI:** 10.1186/s12936-021-03805-z

**Published:** 2021-06-29

**Authors:** Eleanor Hutchinson, Susan Nayiga, Christine Nabirye, Lilian Taaka, Nelli Westercamp, Alexander K. Rowe, Sarah G. Staedke

**Affiliations:** 1 London School of Hygiene & Tropical Medicine, Keppel Street, London, WC1E 7HT UK; 2 Infectious Diseases Research Collaboration, 2C Nakasero Hill Road, Kampala, Uganda; 3 Malaria Branch, Division of Parasitic Diseases and Malaria, Centers for Disease Control and Prevention, 1600 Clifton Road, Atlanta, GA 30333 USA

**Keywords:** Collaborative improvement, Quality improvement, Health Management Information System (HMIS), Malaria, Surveillance, Qualitative, Uganda, Public sector, Data quality

## Abstract

**Background:**

Demand for high-quality surveillance data for malaria, and other diseases, is greater than ever before. In Uganda, the primary source of malaria surveillance data is the Health Management Information System (HMIS). However, HMIS data may be incomplete, inaccurate or delayed. Collaborative improvement (CI) is a quality improvement intervention developed in high-income countries, which has been advocated for low-resource settings. In Kayunga, Uganda, a pilot study of CI was conducted in five public health centres, documenting a positive effect on the quality of HMIS and malaria surveillance data. A qualitative evaluation was conducted concurrently to investigate the mechanisms of effect and unintended consequences of the intervention, aiming to inform future implementation of CI.

**Methods:**

The study intervention targeted health workers, including brief in-service training, plus CI with ‘plan-do-study-act’ (PDSA) cycles emphasizing self-reflection and group action, periodic learning sessions, and coaching from a CI mentor. Health workers collected data on standard HMIS out-patient registers. The qualitative evaluation (July 2015 to September 2016) included ethnographic observations at each health centre (over 12–14 weeks), in-depth interviews with health workers and stakeholders (n = 20), and focus group discussions with health workers (n = 6).

**Results:**

The results suggest that the intervention did facilitate improvement in data quality, but through unexpected mechanisms. The CI intervention was implemented as planned, but the PDSA cycles were driven largely by the CI mentor, not the health workers. In this context, characterized by a rigid hierarchy within the health system of limited culture of self-reflection and inadequate training and supervision, CI became an effective form of high-quality training with frequent supervisory visits. Health workers appeared motivated to improve data collection habits by their loyalty to the CI mentor and the potential for economic benefits, rather than a desire for self-improvement.

**Conclusions:**

CI is a promising method of quality improvement and could have a positive impact on malaria surveillance data. However, successful scale-up of CI in similar settings may require deployment of highly skilled mentors. Further research, focusing on the effectiveness of ‘real world’ mentors using robust study designs, will be required to determine whether CI can be translated effectively and sustainably to low-resource settings.

**Supplementary Information:**

The online version contains supplementary material available at 10.1186/s12936-021-03805-z.

## Background

Transforming malaria surveillance into a core intervention is one of the three pillars of the World Health Organization (WHO) Global Technical Strategy for Malaria (2016–2030), aiming to accelerate progress toward elimination [1]. In Uganda and elsewhere in Africa, data collected at health centres through the Health Management Information System (HMIS) form the backbone of malaria surveillance, and are vital for monitoring disease burden, assessing intervention coverage and impact, and guiding decision-making for programmes and policies. However, the potential limitations of HMIS data are well described [2–4]. In Uganda, it has been estimated that less than 30% of malaria cases are captured in health facilities by HMIS [5]. In addition, such data may be inaccurate, incomplete or delayed, which limits the validity and utility of HMIS surveillance data [6] and may impact negatively on patient care.

Multiple strategies to improve the quality and management of surveillance data have been applied [7–10]. One quality improvement approach, known as ‘collaborative improvement’ (CI), was developed by the Institute for Healthcare Improvement in the mid-1990s [11]. Through CI, health care teams focus on group problem-solving through the application of continuous techniques, known as ‘plan-do-study-act’ (PDSA) cycles. CI encourages participants to reflect on their practices, identify areas for improvement, apply changes, and then assess the impact of these changes on specific indicators. CI approaches have been applied widely in high-income countries [11, 12], and are advocated for low-resource settings [13–15]. The US Government has become a major supporter of quality improvement initiatives for health care, including CI, funding multiple programmes in the health sector over the last decade [16].

Although studies suggest that CI is a promising approach for low- and middle-income (LMIC) settings [13, 14, 17], few rigorously designed, comparative studies of CI have been conducted [18–20], and available data are limited [12, 21]. Moreover, CI has not been widely applied to quality improvement of HMIS surveillance data; and there are gaps in understanding how CI, a complex social intervention, works to change behaviour and improve outcomes [12, 16]. Specifically, the following areas of CI and other quality improvement initiatives are understudied: documentation of the study context to help audiences appreciate the generalizability (and limits to generalizability) of the results; description of the process of implementing the CI intervention; clarification of the mechanisms of action; and, exploration of unintended consequences of interventions, both positive and negative.

In 2015–16, a pilot study was conducted to evaluate the effectiveness, feasibility and cost of implementing CI to improve the quality of malaria surveillance data collected in five public health centres in Kayunga, Uganda. The intervention incorporated brief, in-service training on best recording and reporting practices, and CI. Alongside the quantitative evaluation, an interpretive, qualitative study aiming to ‘open the black box’ of CI and address key knowledge gaps was conducted. The aims were: (1) to describe the context in which, and the processes through which, the CI intervention effected change; (2) to identify any factors that support or undermine CI; and, (3) to investigate for any unintended consequences of the CI intervention.

## Methods

### Study site

**Fig. 1:**
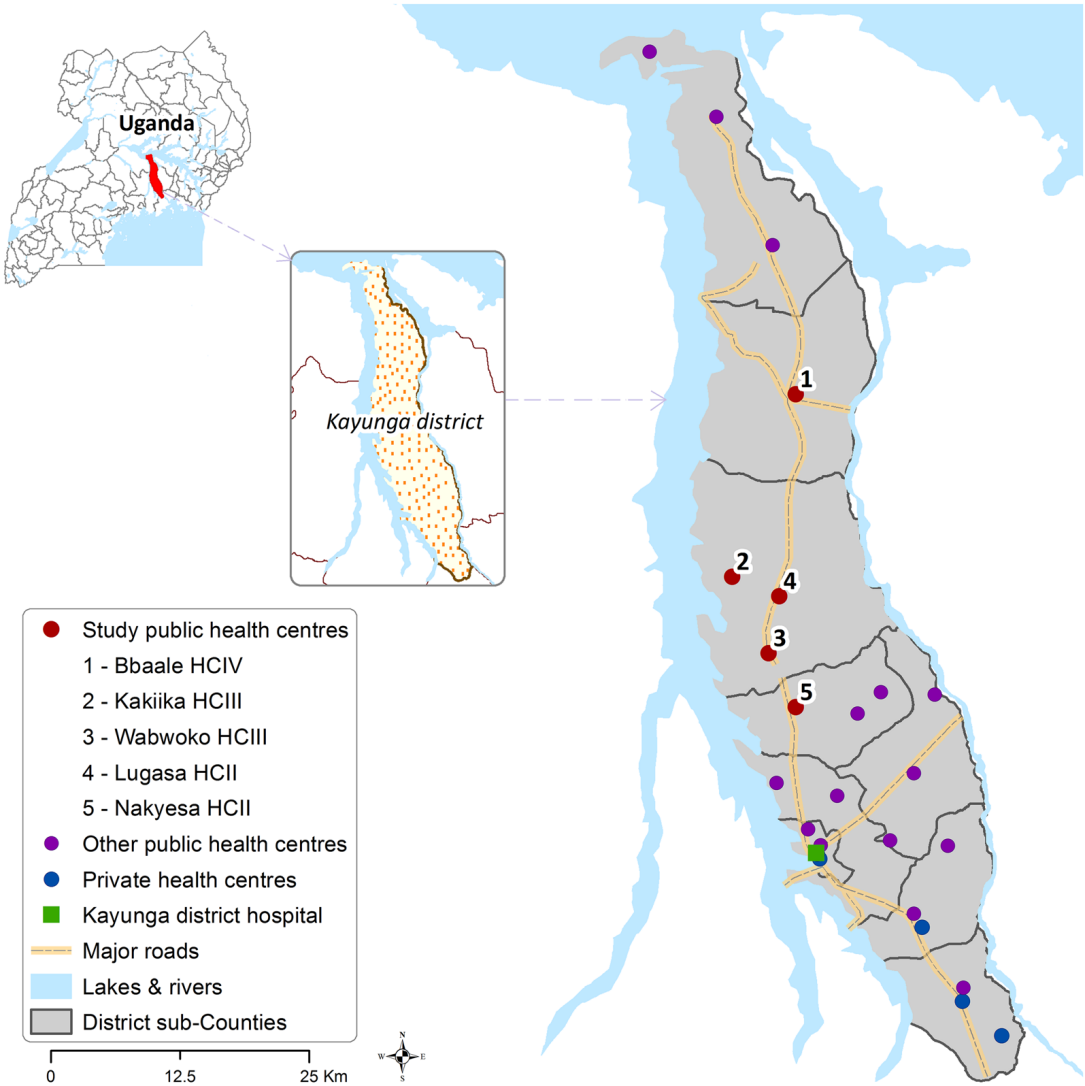
Map of the study area in Kayunga district, Uganda, including the location of private and public (government-run) health centres. HC: health centre; HCII: level II health centre; HCIII: level III health centre; HCIV: level IV health centre

The pilot study was conducted in Kayunga District, Uganda, an area of high malaria transmission [22], which had not previously participated in externally funded, quality improvement initiatives (Fig. 1). Of the 24 public health centres in the district, five within the same hierarchical structure were purposively selected for study participation, with input from the district health team (Table 1): two level II health centres (HC IIs), which serve approximately 4000 to 5500 residents and generally have no laboratory facilities; two level III health centres (HC IIIs), which serve 40,000 to 50,000 residents and are expected to have a functioning laboratory and maternity services; and, one level IV health centre (HC IV), which serves a health sub-district (130,000 residents) and is designed to function as a small hospital serving outpatients and inpatients.

**Table 1:** Basic characteristics of the participating health centres

Name	Number of buildings	Number of rooms	Number of staff members currently stationed at health centre	Number of vacant staff positions	Services offered	Shift system in place?	Village health team members
HC II a	1	4	4	3	Out-patient department, antenatal, family planning, immunization	No	1^‡^
HC II b	1	4	4	3	Out-patient department, antenatal, family planning, immunization	No	10
HC III a	2	12^†^	18	0	Out-patient department, antenatal, family planning, immunization, laboratory, anti-retroviral treatment, limited in-patient department	Yes	6
HC III b	2	16	16	2	Out-patient department, antenatal, family planning, immunization, laboratory, anti-retroviral treatment, limited in-patient department	Yes	5
HC IV	4	35*	36	13	Out-patient department, antenatal, family planning, immunization, laboratory, HIV testing & counselling, in-patient department	Yes	12

^‡^ In HC II a, there were originally 3 active VHTs, however 2 were dismissed, leaving only 1 VHT

^†^ In HC III a, the following rooms are available: antiretroviral clinic = 2 rooms plus a waiting area, outpatient department = 4, maternity = 5, with one room being used as a staff house

* In HCIV, the following rooms are available: antiretroviral clinic = 9, outpatient department = 6, maternity = 8, in-patient = 7; operating theatre = 5

### Study design

**Fig. 2:**
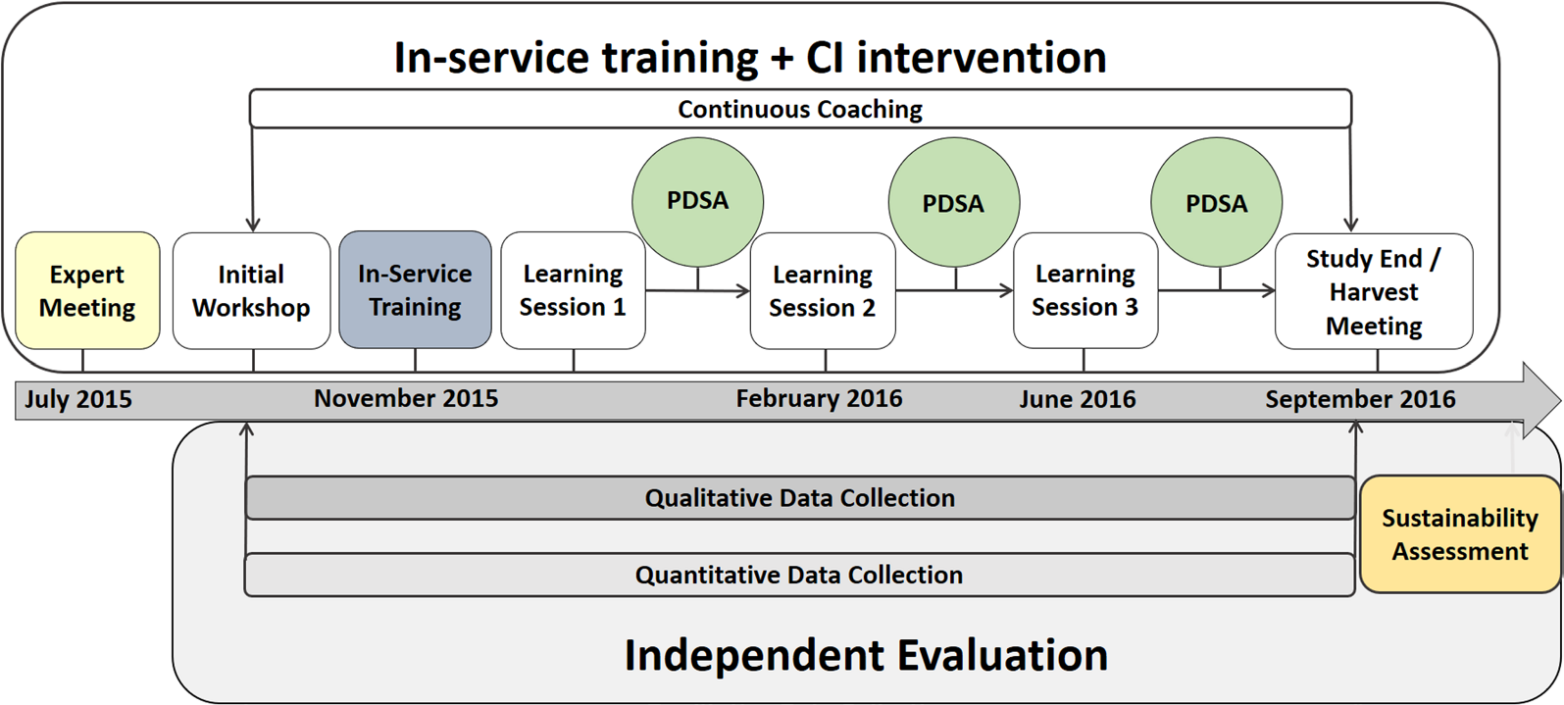
Trial schema, outlining the timelines and study activities, including the components of the intervention and evaluation. CI: collaborative improvement; PDSA: plan-do-study-act

The pilot was designed to evaluate the impact of delivering the study intervention at a cohort of health centres over approximately 1 year (Fig. 2, Additional file 1), using quantitative, qualitative and economic methods. Of note, CI networks typically include 10–100 facilities, but only five health centres were targeted in this pilot study as a proof of concept. The CI intervention and the evaluation were carried out independently by different partners. The results of the quantitative and economic evaluations, as well as the qualitative evaluation of the relationship between the care-giving and data collection, are reported separately [23, 24].

The study intervention consisted of brief in-service training on the HMIS out-patient department (OPD) register and good data collection practices for all health workers, and a quality improvement intervention that followed the CI methodology. In 2015, prior to the onset of the study, the Uganda Ministry of Health introduced an expanded HMIS OPD register. The new OPD register doubled the amount of data collected, increasing the number of indicators from 14 to 31. The new register captured additional malaria-related data (including history of fever, temperature and malaria test result), and demanded the use of technology (thermometers to measure temperature, tapes to measure mid-upper arm circumference, laboratory equipment to measure blood glucose, and calculators to measure body mass index). For the CI component, health workers were selected from each health centre by a senior member of staff to take part in a CI team, (ranging from 4 to 8 members). The CI team participated in learning sessions and coaching visits and were expected to keep a CI journal during the intervening action period, which constituted the PDSA cycles (Table 2, Additional file 1). The CI team were all health workers and no Village Health Team members were invited to the learning sessions or coaching visits.

**Table 2:** The elements and process of the CI intervention

Component	Length of component	Timing	Location	Target audience	Focus	Per diem paid	Overall success
In-service training on completing registers	Half day, 3–4 h	Prior to the start of intervention (4 health centres). During first week of intervention (1 health centre)	All 5 health centres	All health workers at health facilities	How to complete HMIS OPD register, laboratory register, and pharmacy register	No	Health worker attendance was high (70–100% of health workers from participating health centres attended). In subsequent interviews, this was often described as the most important element in the intervention
Initial workshop	Half day, 3–4 h	At the start of intervention	Kampala hotel	District health officials, members of collaborative improvement (CI) team at health centres, intervention team	Introduce study and CI methodology to health workers, create district-wide awareness of activities and support for project, present data on current practices	140,000 UGX (US$40)	Health worker attendance was high (70–100% of health workers from participating health centres attended). Considerable debate emerged about source of poor quality of data. District officials blamed health workers and poor recording practices. Health workers expressed frustration at overwhelming amount of data that they were asked to collect
Learning sessions	3 sessions each 2 days long	At start, at 3 months and at 6 months	District hotel (1); Kampala hotel (2)	Health workers who were members of the CI teams	Formulation of strategies to: 1. Improve data completeness in OPD register (first session) 2. Improving accuracy and concordance between data sources (second session) 3. Improving malaria test and treatment indicators (third session)	140,000 UGX (US$40)	Health worker attendance was high (100% of those invited attended or sent a substitute). Health workers agreed to be involved in the project, but did not grasp the methodology sufficiently to be able to describe it by the end of the intervention
Action periods: Plan, do, study, act (PDSA) cycles	3 action periods of 2–3 months to implement changes from learning sessions	Throughout project	All 5 health centres	Health workers who were members of the CI teams were expected to call meetings to reflect and enact changes, CI mentor who visited health centres to support team members	For health workers to implement changes identified at learning sessions, propose and pilot changes to address problems, collect data to document impact of the changes and decide whether to maintain the change. These are known as PDSA cycles. The CI mentor would arrive spontaneously to conduct these sessions	4000 UGX (US$1.10)	Whilst health workers were expected to lead these sessions, it was the CI mentor who pushed forward action, helping health workers to reflect on the learning sessions, complete project documentation, identify problems, and prompt them to make decisions about the actions that they should take. Initially, the CI mentor also extracted all the data that was expected to underpin reflection, but health workers undertook this towards the end of the intervention. The CI mentor guided health workers to focus on the root of their problems. No spontaneous meetings were observed, and no health worker was observed completing project forms outside meetings with the mentor. Most activity occurred following second learning session
Harvest meeting	2 days	At the end of project	Kampala hotel	Health workers who were members of CI teams	To share lessons learned from the project with participating health workers	140,000 UGX (US$40)	Health worker attendance high (100% of those invited attended or sent a substitute). Health workers found presenting the data from their health facility difficult. Few could describe the theory behind CI or the PDSA cycles. The mentor had to support the health workers so that they could evaluate their data, document and rank the importance of changes made

While meetings with mentors and project staff (the learning sessions and coaching visits) happened periodically and never more than once a month, the overall approach was expected to re-shape daily life in the health facilities. In accordance with the training and the programme, health workers were expected reflect on their practice in journals, to meet regularly to discuss progress around data collection and changes that they had implemented to improve practice. These reflections were then discussed during the coaching visits and learning sessions. At the learning sessions, health workers would also identify new ways to improve data collection in their facilities.

The objective of the qualitative research was to provide a rigorous analysis of CI and the practices associated with the intervention. The data was triangulated by drawing on ethnographic observations and informal discussions, in-depth interviews with individual health workers, and focus group discussions (FGDs) to ascertain group interpretations.

### Ethnographic observations

The ethnographic fieldwork was conducted by the qualitative team of three local social scientists fluent in Luganda, overseen by an anthropologist based overseas who made three visits to Uganda during the study. Ethnographic observations were conducted at each of the five health centres for 12–14 weeks over the 9-month evaluation period. Each social scientist was attached to one (HC IV) or two (HC II and HC III) health centres for the research period. Observations of everyday activities and practices, and CI activities (coaching visits and PDSA cycles), were carried out by the same social scientist throughout the study. HC IIs and HC IIIs were observed for 2 days per week. Observations at the HC IV were carried out for 3–4 days per week.

The fieldwork began with unstructured general observations [25], during which the health centre buildings and movements of patients and health workers were mapped. Subsequently, 5 weeks of structured observations were conducted at the health centres. While the fieldworkers were encouraged to follow particular events as they unfolded, the main focus was on the individuals providing and seeking care, and the ways in which the CI study re-shaped these relationships and the discussions around the CI components and goals among staff. Activities included shadowing patients and health workers, observing patient examinations and the process of data collection. Following this, the team spent a single week at a time on a regular basis in the health centres to observe the impact of the CI intervention. Brief field notes and sketches were written by hand at the health centres and then expanded upon when field notes were written up by the respective social scientists.

### In-depth interviews

Twenty in-depth interviews were conducted by the qualitative team after the ethnographic fieldwork had been completed, including 15 interviews with health workers taking part in the CI teams and 3 with district officials, focusing on data collection and use at the health centres and the CI intervention, and 2 interviews with national stakeholders, focusing on data collection and quality, changes in data collection tools over time, and themes identified in ethnographic observations (the importance and function of data collection in the health facility, relationship between care-giving and data collection, tensions between staff involved and those outside the CI project, the relationship between the CI mentor and the staff). Written informed consent was obtained from participants. Interviews were conducted in English, recorded and transcribed verbatim.

### Focus group discussions

At baseline, three FGDs were conducted by the qualitative team at an off-site location in Kayunga District with a total of 36 health workers from the five health centres, including senior health workers (clinical officers), middle-ranking clinicians, and lower-ranking staff (data entry clerks). The FGDs were stratified by health-worker cadre and aimed to assess the collection and use of data at the health centres, and how health workers learned about the health situation in the community. Another three FGDs were held with 31 health workers in total to explore health-worker experiences with the CI intervention, and sustainability of the changes made at the health centres. Written informed consent was obtained from all FGD participants. The FGDs were conducted in English, apart from the FGD held with lower-cadre health workers, which was conducted in the local language (Luganda). The FGDs were transcribed and when required, translated into English using a meaning-based approach [26].

### Data analysis

The data were analysed using an interpretive, iterative approach which included both theory and data-driven analysis. The fieldwork notes were shared with the senior anthropologist and analysed in an on-going, iterative fashion, and findings from the ethnographic fieldwork were discussed during weekly team meetings. After the observations, the notes were re-analysed thematically by hand. Key findings were mapped in relation to the various elements of CI so that observed changes in practice at the health centres could be associated with elements of CI.

The transcripts of the interviews and FGDs were read and reviewed by the social scientists and senior anthropologist as they were completed. Questions in the topic guide were modified if necessary, based on the responses. Transcripts were also coded in Nvivo qualitative data coding software Version 10 (QSR International), using a mix of pre-defined codes drawn from the ethnographic data and theory but also data-generated themes.

## Results

### The elements and process of CI implementation

The fieldwork was carried out from July 2015 to September 2016 (Fig. 2). Detailed descriptions of the study intervention and field activities are presented in Table 2 and Additional file 1. The results of the quantitative evaluation documented a substantial improvement in data completeness following the introduction of CI, suggesting that the intervention was successful [23]. Overall, the results of the qualitative evaluation suggest that the initial in-service training session was a key component of the study intervention. Health-worker attendance at the in-service training, learning sessions and harvest meeting was high, demonstrating that health workers were active participants of the process. However, the PDSA cycles seem to have been less effective. Health workers did not use their CI journals routinely and meetings were convened by the CI mentor during his visits to the health centres, rather than being organized spontaneously by health workers, suggesting that the CI intervention presented challenges to health workers.

### Local challenges to the CI intervention

The combination of CI and the new OPD registers led to increased work. To complete the new OPD registers, which were introduced just before the CI intervention and included substantially more variables, health workers were required to expand the patient history, carry out additional tests, use different technologies, and record more data. This occurred in a context in which health workers already bore a heavy data collection burden. As one district official reported:

*“Interviewer: Can you tell me about the data collected from patients at the health centres?
*

*Respondent: What kind of data? (laughs) A lot of data is collected there. A lot of data is collected at the centre. There are patient data. We have other data like the infrastructure data, the inventory data, the financial data, yeah. There is patient data from all diseases we see.”* (In-depth interview, district official).

In this context, health workers described the introduction of new procedures for the CI intervention as challenging, adding new pressures to their already busy workdays. The ease of incorporating CI appeared to be influenced by the size of the health centre. In the smaller HC IIs, services are more limited and health workers frequently multitask, taking responsibility for all aspects of patient care, including clinical evaluations, testing (for malaria) and dispensing medications, as well as completing OPD registers. In these spaces, incorporating CI and improving data collection appeared relatively straightforward. However, in the larger HC IIIs and HC IV, patients travel through more complex pathways, navigating the clinical, laboratory and pharmacy services. Health workers also move through the health centres over the course of the day, shifting tasks and working in different spaces. In these complex settings, introducing CI came with considerable operational changes and substantial modifications had to be made to triage, to the flow of patients through the health centre (with additional stopping points added), to the location of the OPD and other registers, and to the chain of custody for patient books (the medical record that remains with the patient).

While the combination of the new OPD register and CI appeared to make considerable demands on the health centre staff, the work involved in collecting data was often considered to have little positive impact on patient care at the health centres. Indeed, some health workers (and their patients) complained that the time patients waited at the health centres extended considerably, negatively impacting on patient satisfaction. That said, over the course of the CI intervention, changes in the perception of the importance of data collection did occur. During the baseline FGDs, health workers were unable to describe the potential uses of the OPD data for their health centre, but during the follow-up interviews with members of the CI teams, data collection was identified as helping health workers to plan, account for medicine use and demonstrate improvements.

As the project developed, this sense of the division between everyday care work that health workers undertook and the work involved in completion of the OPD register and CI was also reflected in their identification of CI and its components as belonging to the CI mentor. They often referred to CI as ‘the mentor’s project’, and the CI journal as ‘the mentor’s book’. Health workers also identified data collection and the CI intervention as being necessary and important for research organizations rather than something that supported their work at the health centre. As one health worker argued:

*“So (Name, CI mentor) has been a good person and he normally calls us when we sit he tells us exactly what we are supposed to do and ideally marry his suggestions because if somebody else comes and does a research here with these registers, they are up to date. Everything is okay, well detailed. They can even follow up somebody, a person who died some time back using these records using the same OPD number until this person went, until the patient got cured or what so that is what (Name, CI mentor) was telling us. And ideally what he has given us it has shown us that in the future we shall also be involved in other researches.”* (In-depth interview, health worker, HC IV).

### CI clashed with hierarchical relationships between health centres

The CI intervention was dependent upon group work and sharing experiences of changing practices of data collection between health centres. Yet, tensions existed between the health centres around the difficulties the HC IV faced in implementing changes, in contrast with the HC IIs and HC IIIs, which found it easier to improve data collection. During informal discussions, senior members of staff, especially at the HC IV, expressed frustration and some embarrassment that their performance was not better than that of the lower-level health centres, which tended to have less well-trained staff and to offer more basic services. While this could be interpreted as setting up a healthy rivalry that might spur on action, during two learning sessions (sessions 2 and 3) and again in the Harvest Meeting, it seemed to impact negatively on the potential for collaboration. Changes that had proved effective in smaller health centres (HC IIs) were rejected outright as not feasible for larger health centres, without critical reflection. For example, when discussing how to manage personal patient records with the new patient pathways:

*“One of the health workers from the HC II proposes that the HC IV takes up the method of putting a mark in the patient book to indicate that it [the patient details] have been entered in the OPD register. The senior member of the HC IV team says that they have tried that before, and it didn’t work. [Later in the meeting] the senior member of the HC IV team tells me that the lower health facility health workers are presenting unrealistic scenarios. The laboratory assistant tells me that it is definitely easier to complete registers in the HC IIIs and IIs.“* (Field notes, learning session 2).

Overall, when coupled with the new OPD register, CI was interpreted as creating more work for health workers in a context of existing data collection demands. CI was often interpreted as belonging to the mentor and of benefit to those conducting research, instead of the health centre and its patients and health workers. CI also clashed with the hierarchies that existed between health centres. Health workers in Health Centre IIIs and IVs, who were often more senior members of staff and viewed their more complex services as superior to those in Health Centre IIs were keen to defend their own practice and criticize those of more junior staff. Nonetheless, the project was considered successful in terms of the uptake of the different elements of CI, and its ability to engender change around data collection practices. Below, the construction of this success and how it was incorporated into everyday practice is analysed.

### Factors underpinning the success of CI

CI was experienced as a high-quality training and supervision. While district health officials had provided training on the new OPD register, many health workers involved in CI reported that they had received little or no training on how to complete the registers. As one health worker described:

The new OPD, nobody trained [us]. They just dumped the book then they said we shall be trained on job. So, that is why for instance when we went for the workshop in Kampala where we scored zero level then we got the ideas after maybe (Name, CI mentor) was telling us how to write in this register because we were not trained. (In-depth interview, Health worker, HC IV)

While some health workers described the in-service training as the most important element of the intervention, for many health workers, the coaching visits seemed to be the key component underpinning change. Health workers described these visits as a form of regular supportive supervision that was otherwise missing in the health system. The mentor, who was very experienced, was highly valued by the health workers; they described him as patient and providing on-going, uncritical support in the face of their daily challenges, and the changes that they sought to make.

### CI brought financial benefits for health workers

While none of the CI team members reported that the financial benefits had influenced their participation in the intervention, the distribution of *per diem* to those who attended meetings and not to those who were working on the CI and the new OPD in the health centres was raised as an important issue during the ethnographic observations, in-depth interviews and FGDs. Officially, *per diem* is paid to cover the costs associated with travel to the workshop and staying overnight. Yet, when it is possible to save this money *per diem* can represent a substantial additional income for health workers, amounting in some instances in this project to around a third of the health worker’s monthly income. The *per diem* provided during the learning sessions were interpreted as payment for the additional work associated with the intervention, and health workers who had not attended these events (and thus received no payments) described themselves (or were described by members of the CI team) as reluctant to undertake the work. As one FGD participant, who had been a member of a CI team explained, this impacted on the ways in which the intervention was rolled out in the health centres following the learning sessions.

Mentoring is not easy because some people are so rigid. You tell someone do this, and that person says, aaah after you [have been] eating money [i.e. getting payments from the project] you are here disturbing us with your work. [Laughter] ‘Ho batwongedde emirimu’ (we have been given more work) so it has not been easy. But slowly, I hope if we are to maintain it [the project] better, I think if those who remained [i.e. have not attended project meetings] are also called in such meetings [as] we are sitting [now], then talked to, things will go [on for] 100 years and years [Laughter] (End of study FGD, higher cadre health workers)

Here, the work associated with the intervention was identified as belonging to the health workers who attended the learning session (“*your work*”), while the key to sustainability of the intervention (its ability to “*go on for 100 years*”) was described as the inclusion of all health workers, ensuring that all receive the financial benefits. This idea that the *per diem* was experienced as payment for work associated with CI was also in evidence during the learning sessions. Below, field notes made during the third learning session suggest that the trainers also see the *per diem* not as recompense for expenses but rather as payment for their active involvement and understanding of CI:

*Trainer X asks for the principles of quality improvement. None of the health workers can remember them. Trainer X says that these people should return the per diem they got during the first learning session.* (Field notes, 3rd learning session)

### CI was carried out by small, committed teams with help from volunteers and patients

During informal conversations and in-depth interviews, health workers described how, prior to the implementation of the intervention, the responsibility for completing the OPD register was shared amongst multiple health workers, particularly at the larger health centres. Once CI was established with the CI teams, who received training and payments, in the higher-level health centres (HC IIIs and HC IV) new divisions of labour emerged between those who took responsibility for data and those who did not. Health workers outside of the CI team continued to express dissatisfaction with the intervention and frustration with the burden of work presented by the new OPD register. In contrast, many of the members of the CI team became attached to the intervention and expressed loyalty to the project and the CI mentor.

While health workers who were not part of the CI team did not champion or push forward improvements in data collection, they were observed to change their practices aiming to improve the completeness of the OPD data. As CI became more embedded in the larger health centres, the additional work involved in improving data quality was also managed by recruiting patients and volunteers to take simple measurements (such as weigh, height and temperature) and to record data in the register. Patients would help with height and weight measurements and village health team members (VHTs) took on a variety of responsibilities that included recording patient details, completing the OPD registers, completing anti-retroviral cards for HIV, triaging patients, screening for tuberculosis, testing for malaria, and dispensing medications.

## Discussion

CI is a quality improvement strategy that has been increasingly advocated for, and applied in, low-resource settings. In this pilot study, CI was applied specifically to improve the quality of malaria surveillance data in Kayunga, Uganda. The results of the quantitative evaluation, which will be reported elsewhere, documented an improvement in data completeness following the introduction of the study intervention [23]. The qualitative evaluation reported here aimed to ‘open the black box’ of CI, to examine the way in which CI was interpreted by health care workers and how it was absorbed into everyday practice. Through ethnographic observations, interviews and FGD some CI activities appeared to be implemented as planned (learning sessions, harvest meeting and coaching visits), but other activities (collection and interpretation of information and indicators related to the CI process, regular facility-level CI team meetings, root cause analysis, and PDSAs) were not generally implemented by health facility staff and were largely accomplished through the efforts of the CI mentor. The fact that health workers were only observed using the CI journal (in which these reflections were expected to be noted) when the mentor visited the centres supports this finding. These results suggest that the CI intervention successfully facilitated change in data quality, but not through the expected mechanisms. Rather than being an internally led intervention, with changes driven by self-reflection and techniques that enable subjects to improve their own performance, as CI is hypothesized to work, the intervention was understood by health workers as an external initiative, which was (highly) valued for the frequent and supportive meetings with the CI mentor and the financial benefits accrued from the project.

In this context, it appears that much of the CI activity in these health centres was facilitated by the relationships formed by the CI mentor with the health workers, and the supportive ‘supervision’ provided by the mentor. These social relations and practices were also underpinned by the exchange of resources that was associated with the additional work carried out to collect the data. Financial compensation and its potential impact on the intervention’s success and sustainability is an important but sensitive topic. The difficulties of exchanging resources in global health programming has been described by others [27]. If health workers are required to travel away from home for training or meetings, defraying the costs of their travel is appropriate and would be expected. In Uganda, salaries of government health workers in Uganda are low (and often inconsistent), ranging from a gross annual salary of approximately US$2400 for a clinical officer or nursing officer, to US$1400 for a records assistant. *Per diem* is commonly paid for attendance at meetings and conferences and is embedded in Ugandan practice, with many health workers relying on *per diem* to top-up their existing salaries. It was not possible to determine whether the CI intervention would have been successful without the extra financial benefits to health workers, and the question of whether any level of compensation can be provided at scale, sustainably, remains.

This study had several limitations. As a pilot study, it only included public health centres, with a specific focus on the impact of CI on the quality of malaria surveillance data. While three levels of health centres were included, these results may not be representative of the reporting practices and the effect of the CI approach of the entire healthcare system in Uganda. That said, these health centres are typical for Uganda in terms of their recording and reporting practices. The aim of the qualitative evaluation was an in-depth exploration of the inner workings of the CI method for improvement of the surveillance data quality; this intent is best realized through a small study, due to the large efforts required to conduct the qualitative work. Additionally, the recall and reporting bias may be present in situations when health care workers are uncomfortable disclosing their truthful opinions about their health centres, their role in the intervention and the intervention itself. The study attempted to minimize this bias by triangulating data from ethnographic observations, FGDs and in-depth interviews. In addition, during the in-depth interviews researchers assured health workers of the confidentiality of their responses and commitment to reporting the results in a manner that would limit the possibility of identification of specific responders.

## Conclusions

CI is a promising approach to quality improvement, and could be applied to strengthen the quality of malaria surveillance data. However, successful scale-up of CI in similar low-resource settings may require deployment of highly skilled mentors and raises questions of how feasible it would be without financial incentives. Overall, the analysis presented here poses questions about the feasibility and sustainability of scaling up CI effectively with intensive mentoring, and the cost-effectiveness of such programmes. This pilot project suggests that supportive supervision, like that provided by the CI mentor in this project, was otherwise lacking in these health centres, but is highly valued and desired by health workers. Further research with robust study designs that includes an in-depth qualitative component and ethnographic observation will be required to determine whether CI is likely to be an effective, sustainable or appropriate intervention to improve the quality of HMIS data collection in low-income settings. These could explore whether investing in non-financial incentives that were of concern to health workers (improvements in their training, mentoring and supportive supervision) could replace financial incentives that were in evidence in this pilot study. Recognizing that the problems of poor salary and widespread use of per diems goes beyond this pilot intervention, future studies could focus, in part, on the effectiveness of CI at scale, when ‘real-world’ mentors are used and could analyse whether if CI projects could respond to the concerns of staff members then they would be taken up without the need for substantial per diem payments.

## Supplementary Information

**Additional file 1.** Study components.

## Data Availability

The qualitative data that support the findings of this study are not publicly available, as this would compromise participant privacy. Participants did not consent to have their interview transcripts made publicly available. However, data may be available from the corresponding author on reasonable request.

## Abbreviations

HMIS: Health Management Information Systems
CI: Collaborative improvement
PDSA: Plan-do-study-act
WHO: World Health Organization
LMIC: Low- and middle-income country
HC: Health centre
OPD: Out-patient department
FGD: Focus group discussion
CDC: Centers for Disease Control and Prevention
VHT: Village health teams
