# Supplementary material for: Opening the ‘black box’ of collaborative improvement: a qualitative evaluation of a pilot intervention to improve quality of malaria surveillance data in public health centres in Uganda
Source: Malar J. 2021 Jun 29;20:289. doi: 10.1186/s12936-021-03805-z (PMC8243860; doi:10.1186/s12936-021-03805-z)
Supplement: Supplementary file 1 — Additional file 1. Study components. [file 12936_2021_3805_MOESM1_ESM.docx]

**Opening the ‘Black Box’ of Collaborative Improvement: A qualitative evaluation of a pilot intervention to improve quality of malaria surveillance data in public health centres in Uganda**

**Additional File 1. Study components**

| **Component** | **Purpose** | **Study partners responsible** | **Location** | **Date** | **Attendees** |
| --- | --- | --- | --- | --- | --- |
| ***Expert meeting*** | A meeting of national and international malaria data quality experts resulting in development of the ‘implementation package’ | Uganda Malaria Surveillance Project (UMSP) | Protea Hotel, Kampala | 12-13 July 2015 | Representatives from the company managing the intervention; US President’s Malaria Initiative (PMI), and US Centers of Disease Control and Prevention (CDC); Uganda Ministry of Health and Uganda Malaria Surveillance Project (UMSP), the University of California, San Francisco (UCSF); London School of Hygiene & Tropical Medicine (LSHTM) and Infectious Disease Research Collaboration (IDRC). |
| ***In-service training*** | To carry out an interactive, site-specific training on best recording and reporting practices. | UMSP | HC III a | 4 November 2015 | Representatives from UMSP, CDC, LSHTM, social scientists observing the meeting, all health workers from the participating health centres (~40 health workers trained) |
|  |  |  | HC III b | 5 November 2015 |  |
|  |  |  | HC II b | 6 November 2015 |  |
|  |  |  | HCIV | 9 November 2015 |  |
|  |  |  | HC II a | 23 November 2015 |  |
| ***Initial workshop*** | To convene the district administration, the facility in-charges, and the representatives of major departments from all the participating centres to introduce the study and the CI methodology, create awareness of study activities in the district and to gain the support from the District Health Office. | Company managing the intervention | Hotel in Kampala | 11 November 2015 | Representatives from Uganda’s National Malaria Control Programme (1), district health officials (5), company managing the intervention, CDC, LSHTM, UMSP, social scientists, and health workers from participating health centres (HC IV: 6, HC III b: 4, HC III a: 4, HCII b: 2, HCII a: 2) |
| ***First learning session*** | To introduce the concept of quality improvement, form the facility-based CI teams and to develop site-specific improvement changes | Company managing the project | Hotel, Kampala | 11-12 November 2015 | Company managing the project, CDC, LSHTM, social scientists, and health workers from participating health centres (HC IV: 6, HC III b: 4, HC III a: 4, HC II b: 2, HC II a: 2) |
| ***Action periods:***  ***Plan, do, study, act (PDSA) cycles*** | To implement PDSA cycles and document the changes and their outcomes by filling out the quality improvement journals and sampling data before and after each change to be recorded in tables and time charts. | Health workers at the five study health centres | At the study health centres | Time between the learning sessions | All health workers from the participating health centres |
| ***1^st^ coaching visit*** | To enhance team’s proficiency with the quality improvement methodology, focusing on how to implement a change, rather than what change to try out. | Company managing the project | HC II b | 23-25 November 2015 | Company managing the project, social scientists, and health workers from the participating health centres |
|  |  |  | HC III a | 24-25 November 2015 |  |
|  |  |  | HC III b | 25 November 2015 |  |
|  |  |  | HC IV | 25 November 2015 |  |
|  |  |  | HC II a | Not observed |  |
| ***2^nd^ coaching visit*** | To enhance team’s proficiency with the quality improvement methodology, focusing on how to implement a change, rather than what change to try out. | Company managing the project | HC III a | 15-16 December 2015 | Company managing the project, social scientists, and health workers from the participating health centres |
|  |  |  | HC II b | 15 December 2015 |  |
|  |  |  | HC III b | 16 December 2015 |  |
|  |  |  | HC IV | Not observed |  |
|  |  |  | HC II a | Not observed |  |
| ***Second learning session*** | To focus on data accuracy, with the aim of improving the recording of malaria case management practices (testing, diagnosing, and treatment practices) | Company managing the project | Hotel in the local district | 9-10 February 2016 | Company managing the project, CDC, LSHTM, social scientists, and selected CI team members from the participating health centres |
| ***3^rd^ coaching session*** | To enhance team’s proficiency with the quality improvement methodology, focusing on how to implement a change, rather than what change to try out. | Company managing the project | HC II b | 16 March 2016 | Company managing the project, social scientists, and health workers from the participating health centres |
|  |  |  | HC IV | 17 March 2016 |  |
|  |  |  | HC III a | 17 March 2016 |  |
|  |  |  | HC III b | Not observed |  |
|  |  |  | HC II a | Not observed |  |
| ***Third learning session*** | To develop a sustainability plan, to maintain the achieved improvements in the longer term. | Company managing the project | Hotel in Kampala | 21-22 June 2016 | Company managing the project, CDC, UMSP, LSHTM, social scientists, district health officials (4), and selected CI team members from the participating health centres (18) |
| ***Harvest meeting*** | To document the changes made by the participating health centres, to rank them in terms of importance and ease of execution, and to compile them into a comprehensive “change package” | Company managing the project | Hotel in Kampala | 14-15 September 2016 | Company managing the project, CDC, UMSP, LSHTM, social scientists, district health officials, and selected CI team members from the participating health centres |
